# A Mitochondrial Disorder in a Middle Age Iranian Patient: Report of a Rare Case

**DOI:** 10.18869/nirp.bcn.8.4.337

**Published:** 2017

**Authors:** Mostafa Almasi, Mohammad Reza Motamed, Masoud Mehrpour, Bahram Haghi-Ashtiani, Fahimeh Haji Akhondi, Yalda Nilipour, Seyed-Mohammad Fereshtehnejad

**Affiliations:** 1. Department of Neurology, Firoozgar Hospital, Iran University of Medical Sciences, Tehran, Iran.; 2. Pediatric Pathology Research Center, Mofid Children Hospital, Shahid Beheshti University of Medical Sciences, Tehran, Iran.; 3. Department of Neurobiology, Care Sciences and Society, Karolinska Institutet, Stockholm, Sweden.

**Keywords:** Mitochondrial disorder, MELAS Syndrome, Middle age

## Abstract

**Introduction:**

Mitochondrial encephalopathy, lactic acidosis, and stroke-like episodes (MELAS) can involve multiple systems and cause stroke-like episodes and status epilepticus.

**Case Presentation:**

A 48-year-old female with history of early fatigability, migraine-type headaches, and bilateral sensory-neural hearing loss presented 3 episodes of serial seizures. On admission she was affected by Wernicke aphasia and, then, right hemiparesis. Investigations showed elevated arterial lactate and ragged red fibers on muscle biopsy.

**Conclusion:**

Though more commonly diagnosed during childhood, some cases of adult-onset MELAS syndrome are reported. This syndrome should be considered in patients with stroke-like events in adults without cerebrovascular risk factors and difficult-to-treat seizures.

## 1. Introduction

One of the metabolic mitochondrial disorders is the syndrome of mitochondrial encephalopathy, lactic acidosis, and stroke-like episodes (MELAS), which represents with multisystem involvement (Yoshida et al., 2013) MELAS is the most common mitochondrial myopathy, yet rare with a prevalence of 0.18 per 100000 (Yatsuga et al., 2012). The most common clinical feature of MELAS syndrome is the stroke-like episodes (Kim et al., 1996); however, other features including muscle weakness, headache, vomiting, short stature, dementia, seizure, cardiomegaly, deafness, and endocrinopathies complete the classic clinical scenario (Chae et al., 2004).

The strokes occur in mitochondrial disorders, including MELAS syndrome, tend not to have a vascular distribution, but rather happen in the occipital lobe (Cohen, 2013). These stroke-like episodes may show a variety of neurological symptoms such as migrainous headaches, homonymous hemianopsia, an altered mental status, and seizures (Pavlakis, Phillips, DiMauro, De Vivo, Rowland, 1984). Seizures may occur either as series or even status epilepticus (Yoshida et al., 2013).

The inheritance of MELAS syndrome is in the maternal line and the disease usually manifests from 2 to 10 years old (Pavlakis et al., 1984). Nevertheless, there are few cases with disease-onset during late adulthood up to the age of 62 (Hsu et al., 2012; Gilchrist, Sikirica, Stopa, & Shanske, 1996; Corr, Gaughan, Moroney, & Looby, 2014). The current report was a rare case with MELAS syndrome with symptoms represented at the age of above 40 years.

## 2. Case Presentation

**Figure 1:**
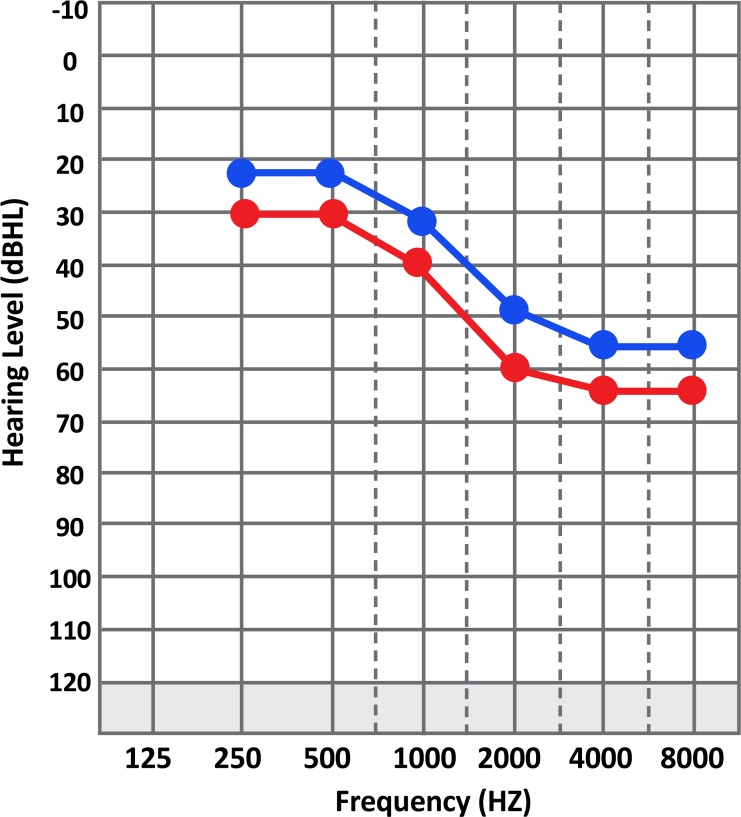
Pure tone audiogram, sensorineural hearing loss (blue line: Air conduction, red line: Bone conduction).

A 48-year-old female admitted to the hospital complaining of 3 times generalized tonic-clonic seizures. The seizures occurred at sleep 2 days before admission and decreased level of consciousness lasted until admission. She was taking sodium valproate since two months ago, when her 1st seizures begun. Her attacks appeared as a series of seizures and the patient had decreased level of consciousness for 4 days following seizures. In her past medical history, she had no developmental problems; however, she had a history of bilateral sensory-neural hearing loss since the age of 35 (Figure 1), also migraine-type headaches and early fatigability in recent years. There was no family history of neurologic diseases. During the 1st physical examination, she obeyed only 1-step orders, had fluent speech with neither fever nor neck rigidity nor optic disc swelling. She had gaze preference to left side, but no weakness or Babinski sign was detected.

Laboratory evaluations demonstrated mild anemia (hemoglobin 11.6 g/dL), elevated erythrocyte sedimentation rate (43 mm), and mild respiratory alkalosis on arterial blood gas analysis. Serum electrolytes, calcium, liver function tests, and creatinine phosphokinase were all normal. Analysis of the cerebrospinal fluids (CSF) was also normal. Initial brain Computed Tomography (CT) scan showed no significant lesion.

**Figure 2:**
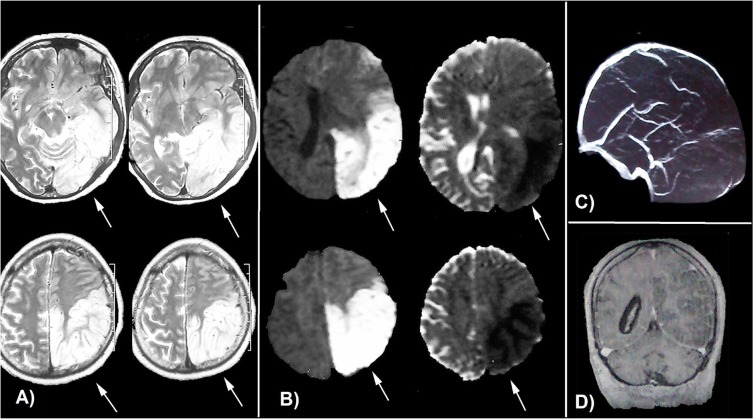
A) Axial t2-weighted brain MRI; hypersignaling in left parietooccipital lobes with mass effect; B) Axial diffusion-weighted MRI and ADC show water restriction in left parietooccipital lobes related to acute ischemia; C) Brain MRV shows no abnormality in dural sinuses; D) Coronal t1-weighted brain MRI with contrast injection; hypo-signality in left parietooccipital lobes with mass effect on lateral ventricle without contrast enhancement. (ADC: Apparent Diffusion Coefficient; MRV: Magnetic Resonance Venography)

At the second day of admission, the patient developed right hemiparesis with muscle force grade 4 of 5, and Wernicke aphasia. New brain CT scan showed mass effect on the left hemisphere. Diffusion-weighted brain Magnetic Resonance Imaging (MRI) showed large t2-hyperintense lesion in the left parietooccipital lobes with falcine, subfalcine, and right tentorial herniations (Figure 2). Due to suspicion to cerebral vein thrombosis, brain MR-venography was performed, which was normal (Figure 2). Mannitol (20%, 300 mL bullous and 150 mL every 4 hours for 24 hours) and dexamethasone (24 mg daily in divided doses) were prescribed.

Additional laboratory tests indicated elevated arterial lactate (24 mg/dL, normal range: 4.5 to 14.5 mg/dL), normal thyroid function tests, antinuclear antibody, anti-cardiolipin antibody, antiphospholipid antibody, rheumatoid factor, and anti-neutrophil cytoplasmic antibody. No cardiac abnormality was observed on electrocardiogram, transthoracic echocardiography, and saline-infusion echocardiography. Results of transcranial ultrasonography and carotid Doppler were normal. Brain conventional angiography showed no arterial occlusion. Electroencephalography (EEG) showed diffuse slow activity with left side sharp waves.

**Figure 3:**
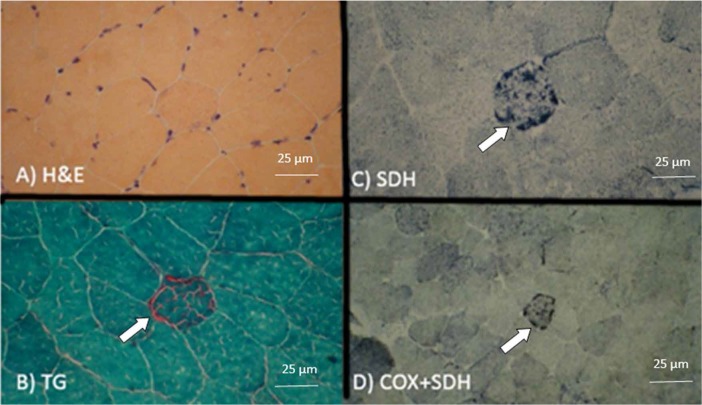
Biopsy specimen from deltoid muscle: A) H & E staining (400×); fiber size variation with few internalized nuclei and cytoplasmic basophilic discoloration; B) Modified TG staining (400×): ragged red fiber; C) & D) Abnormal peripheral proliferation of mitochondria in SDH staining and no detection of COX negative fiber in combined COX and SDH staining (400×). (H & E: Hematoxylin and Eosin; SDH: Succinate Dehydrogenase; TG : Gomori Trichrome; COX: Cytochrome C Oxidase)

Biopsy specimen was obtained from left deltoid muscle to rule out mitochondrial disorders. Fresh muscle sample was frozen in isopentane cooled in liquid nitrogen. It showed slight fiber-size variation with few internalized nuclei, and some fibers with slight cytoplasmic basophilic discoloration (Figure 3-A). Histochemical studies on frozen muscle sample showed multiple ragged red fibers (the modified Gomori trichrome stain, Figure 3-B). Abnormal peripheral proliferation of mitochondria (succinate dehydrogenase stain) (Figure 3-C) was observed in multiple fibers. There was no COX (combined cyto-chrome C oxidase and succinic dehydrogenase stain) negative fiber (Figure 3-D). Finally, the diagnosis of mitochondrial myopathy with COX positive ragged red fibers was suggested, which was compatible with clinical diagnosis of MELAS syndrome.

Level of consciousness and aphasia were improved on the 4^th^ and 12^th^ days of admission, respectively. She was discharged on the 22^nd^ day of admission with mild hemiparesis under treatment with coenzyme-Q 10, L-carnitine, and multivitamins. After 6 months of follow-up, she had neither paresis nor aphasia, and her seizures were controlled by levetiracetam.

## 3. Discussion

The reported patient presented serial seizures followed by hemiparesis and aphasia. Brain imaging showed an ischemic lesion in the left hemisphere predominantly located in occipital lobe. Her aphasia and hemiparesis markedly improved with conservative treatments within 2 weeks. Stroke-like episodes are the most common clinical features of MELAS syndrome (Kim et al., 1996). In addition to hemiparesis, these episodes may present with other neurologic symptoms including migrainous headaches, homonymous hemianopsia, an altered mental status, and seizures (Pavlakis et al., 1984). Seizures, especially status epilepticus or epilepsia partialis continua, which are difficult-to-treat, raise the possibility of mitochondrial disorders (Bindoff & Engelsen, 2012).

It is more plausible to consider MELAS as a potential diagnosis in patients who have experienced worsened seizure activity under treatment with valproic acid (Hsu et al., 2012). Yoshida et al., reported a patient with 2 episodes of status epilepticus (similar to the patient in the current report) and suggested that a more protracted course of seizures with a persistent altered mental status was concomitant with larger brain lesions (Yoshida et al., 2013). On the other hand, stroke-like episodes as the most common clinical features, and the corresponding changes in brain imaging are very helpful to guide clinicians to correctly diagnose MELAS syndrome (Pauli, Zarzycki, Krzysztalowski, & Walecka, 2013).

Another important diagnostic procedure in mitochondrial disorders is muscle biopsy. Microscopic changes, such as description of the “ragged-red fibers”, which are observed in muscle specimens following staining with modified Gomori trichome, are often associated with mitochondrial myopathies (Cohen, 2013). These disabled mitochondria accumulate in the muscles and manifest as described.

Although the basic pathophysiology of stroke-like episodes is unknown, alterations in nitric oxide homeostasis and over-reduction/oxidative stress are recommended as the 2 leading mechanisms (Iizuka & Sakai, 2010). In clinical practice, partial effectiveness of immediate and long-term therapy with L-arginine and L-citrulline to prevent subsequent strokes (Jobgen, Fried, Fu, Meininger, Wu, 2006) suggests that the role of nitric oxide metabolism is very important.

Most of the cases with MELAS syndrome manifest their 1st features when they are 2 to 10 years old (Pavlakis et al., 1984). In recent years, there were some rare case reports presenting MELAS syndrome during adulthood (Hsu et al., 2012; Gilchrist et al., 1996; Corr et al., 2014). Vrettou et al. (2013) reported a 56-year-old patient presenting with status epilepticus and hypo-density in the left temporal lobe in brain CT scan. Also, a Taiwanese patient was reported with stroke-like episodes at the age of 62 (Li et al., 2007). Mitochondrial disorders progress at different rates (Cohen, 2013) and it could be possible that some factors such as nutrition, physical and behavioral activities, contact with oxidative stressors, and anti-oxidants may cause delayed evolution of their symptoms.

In conclusion, MELAS syndrome is one of the mitochondrial disorders with a variety of symptoms and should be considered in adult patients with stroke-like events without cerebrovascular risk factors and difficult-to-treat seizures.
